# Dung‐visiting beetle diversity is mainly affected by land use, while community specialization is driven by climate

**DOI:** 10.1002/ece3.9386

**Published:** 2022-10-08

**Authors:** Jana Englmeier, Christian von Hoermann, Daniel Rieker, Marc Eric Benbow, Caryl Benjamin, Ute Fricke, Cristina Ganuza, Maria Haensel, Tomáš Lackner, Oliver Mitesser, Sarah Redlich, Rebekka Riebl, Sandra Rojas‐Botero, Thomas Rummler, Jörg‐Alfred Salamon, David Sommer, Ingolf Steffan‐Dewenter, Cynthia Tobisch, Johannes Uhler, Lars Uphus, Jie Zhang, Jörg Müller

**Affiliations:** ^1^ Field Station Fabrikschleichach, Department of Animal Ecology and Tropical Biology Julius‐Maximilians‐University Würzburg Rauhenebrach Germany; ^2^ Department of Conservation and Research Bavarian Forest National Park Grafenau Germany; ^3^ Department of Conservation Biology Goethe University Frankfurt Frankfurt am Main Germany; ^4^ AgBioResearch and Ecology, Evolution and Behavior Program, Department of Entomology Michigan State University East Lansing Michigan USA; ^5^ AgBioResearch and Ecology, Evolution and Behavior Program, Department of Osteopathic Specialties Michigan State University East Lansing Michigan USA; ^6^ TUM School of Life Sciences, Ecoclimatology Technical University of Munich Freising Germany; ^7^ Department of Animal Ecology and Tropical Biology, Biocenter Julius‐Maximilians‐University Würzburg Würzburg Germany; ^8^ Professorship of Ecological Services, Bayreuth Center of Ecology and Environmental Research (BayCEER) University of Bayreuth Bayreuth Germany; ^9^ Chair of Restoration Ecology, School of Life Sciences Technical University of Munich Freising Germany; ^10^ Institute of Geography University of Augsburg Augsburg Germany; ^11^ Institute of Ecology and Evolution & Field Station Schapen University of Veterinary Medicine Hannover Hannover Germany; ^12^ Department of Zoology, Faculty of Science Charles University Praha‐2 Czech Republic; ^13^ Department of Ecology, Faculty of Environmental Sciences Czech University of Life Sciences Prague Praha‐Suchdol Czech Republic; ^14^ Institute of Ecology and Landscape Weihenstephan‐Triesdorf University of Applied Sciences Freising Germany

**Keywords:** coleoptera, coprophagous beetles, decomposition, global change, hill numbers, network analysis

## Abstract

Dung beetles are important actors in the self‐regulation of ecosystems by driving nutrient cycling, bioturbation, and pest suppression. Urbanization and the sprawl of agricultural areas, however, destroy natural habitats and may threaten dung beetle diversity. In addition, climate change may cause shifts in geographical distribution and community composition. We used a space‐for‐time approach to test the effects of land use and climate on α‐diversity, local community specialization (*H*_2_′) on dung resources, and γ‐diversity of dung‐visiting beetles. For this, we used pitfall traps baited with four different dung types at 115 study sites, distributed over a spatial extent of 300 km × 300 km and 1000 m in elevation. Study sites were established in four local land‐use types: forests, grasslands, arable sites, and settlements, embedded in near‐natural, agricultural, or urban landscapes. Our results show that abundance and species density of dung‐visiting beetles were negatively affected by agricultural land use at both spatial scales, whereas γ‐diversity at the local scale was negatively affected by settlements and on a landscape scale equally by agricultural and urban land use. Increasing precipitation diminished dung‐visiting beetle abundance, and higher temperatures reduced community specialization on dung types and γ‐diversity. These results indicate that intensive land use and high temperatures may cause a loss in dung‐visiting beetle diversity and alter community networks. A decrease in dung‐visiting beetle diversity may disturb decomposition processes at both local and landscape scales and alter ecosystem functioning, which may lead to drastic ecological and economic damage.

## Graphical abstract

This research article investigated land‐use and climate effects on the alpha‐diversity, local community specialization, and gamma‐diversity of dung beetles. We found that alpha‐ and gamma‐diversity were negatively affected by agricultural and urban land use, on a local and regional scale. Community specialization, however, increased with decreasing temperatures.

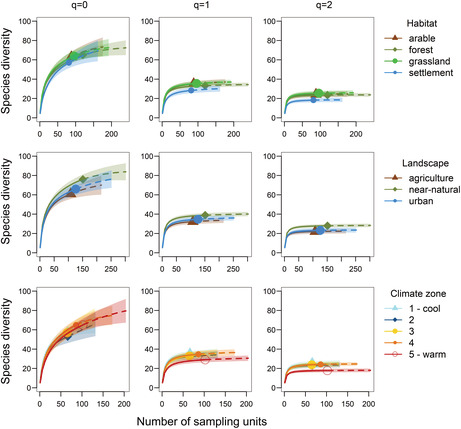

## 1 INTRODUCTION

One fundamental yet often overlooked process for terrestrial ecosystem functions is the decomposition of vertebrate dung by beetles (Hanski & Cambefort, 1991; Pecenka & Lundgren, 2018). This functional group is frequently used as a bioindicator for habitat quality and conversion (McGeoch et al., 2002) and is particularly sensitive to land‐use intensity and climate (Carpaneto et al., 2007; Gardner et al., 2008; Menéndez et al., 2014; Sánchez‐Bayo & Wyckhuys, 2019).

Land‐use intensification, as a consequence of the constantly growing global human population (Seto et al., 2011), comes along with more intensive management techniques and the transformation of natural habitats to agricultural and urban areas, which may negatively affect dung beetle abundances (Carpaneto et al., 2007; Gardner et al., 2008; Sánchez‐Bayo & Wyckhuys, 2019). Additionally, dung beetles are faced with climate change. Beetles are poikilothermic, and their feeding activities and population dynamics, e.g., population growth, are sensitive to temperature (Frazier et al., 2006). Increasing temperatures may contribute to thermal stress that can affect their phenology, community structure, and ecosystem functions (Angilletta, 2009; Barton & Bump, 2019; Graham & Grimm, 1990; Warren et al., 2013). Even a change in the geographical distribution of dung beetles, e.g., to higher elevational ranges, has been suggested as a consequence of long‐term climate change (Menéndez et al., 2014). Since climate change is often associated with rising temperatures and changes in precipitation patterns (Collins et al., 2013), it is vital to investigate the effects of both temperature and precipitation on dung beetle assemblages.

In general, a decline in dung beetles not only results in lower decomposition rates of dead organic material (necromass) (Frank, Hülsmann, et al., 2017). It potentially leads to shifts in the self‐regulation of ecosystems, since dung beetles contribute to nutrient cycling, soil aeration, secondary seed dispersion, and parasite suppression (Evans et al., 2019; Nichols et al., 2008). For example, a decline in dung beetles is likely to cause fouling of grasslands and an increase in livestock parasite and pest species, which may have drastic economic consequences (Castle & MacDaid, 1972; Losey & Vaughan, 2006).

Changes in abundance, number of species, and community composition, e.g., by habitat loss, may affect community networks and stability (Neff et al., 2021; Spiesman & Inouye, 2013). Climate, moreover, might also moderate the structure and dynamics of networks (Classen et al., 2020). Community networks can be described by the structure and density of interaction links, and allow, inter alia, drawing conclusions about the specialization of individual species or communities (Neff et al., 2021; Newman & Girvan, 2004; Spiesman & Inouye, 2013), for instance about the specialization of dung beetles on dung types. Network stability depends on the connectivity, the number of interactions in a network, and the network size. Therefore, species‐rich networks can enhance community stability (Neff et al., 2021; Spiesman & Inouye, 2013) and resilience to the loss of single species through climate or land‐use change.

Although dung beetles are known to be good bioindicators of ecosystem health (McGeoch et al., 2002), most studies on insect networks focus on plant‐pollinator interactions and neglect dung beetle networks (but see Frank et al., 2018). In addition, most research on dung beetles has hitherto focused on forest and agricultural systems (Carpaneto et al., 2007; Frank, Hülsmann, et al., 2017; von Hoermann et al., 2020; Weithmann et al., 2020).

This study is among the first to investigate dung beetle assemblages across a large range of typical land‐use types in temperate regions, ranging from near‐natural landscapes to highly disturbed agricultural and urban landscapes, and along a large climate gradient.

Using a space‐for‐time approach with independent climate and land‐use gradients, we investigated α‐diversity as an abundance of dung‐visiting beetles, species density (sensu Gotelli & Colwell, 2001), and species richness (sensu Gotelli & Colwell, 2001), local community specialization on dung resources, and γ‐diversity as an indicator for community homogenization. We used a fully crossed design along both land‐use and climate gradients at local (habitat) and regional (landscape) scales. Specifically, we addressed the following research questions:

1. Do local habitat and regional landscape types affect α‐diversity, local community specialization on dung types, and γ‐diversity of dung‐visiting beetles?
2. Do temperature and precipitation affect α‐diversity, local community specialization on dung types, and γ‐diversity of dung‐visiting beetles?

## 2 MATERIALS AND METHODS

### 2.1 Study sites

This space‐for‐time study was conducted on 115 study sites, embedded in 44 study regions and along two independent gradients of land‐use intensity and climate in southeast Germany (Bavaria) (Redlich et al., 2022) (Figures S1 and S2). Each of the 44 study regions (à 5.8 km × 5.8 km) was assigned to the dominant regional landscape type (near‐natural, agricultural, urban). The regional landscape types consisted of 16 near‐natural landscapes (defined as >85% near‐natural vegetation including a minimum of 50% forest), 15 agricultural landscapes (>40% arable land and managed grassland), and 13 urban landscapes (>14% housing, industry, and traffic infrastructure).

Within the 44 study regions, 115 study sites were embedded and distinguished in four habitat types (forest, grassland, arable field, and settlement). Within each study region, the three most dominant local land‐use types (habitats) out of the forest, grassland, arable field, and settlements were selected for establishing study sites (3 m × 30 m). Habitats were represented as 36 forest sites (forest clearings), 28 grassland sites (meadows), 27 arable fields (crop field margins), and 24 settlements (green spaces within settlements or cities).

Study regions covered five climatic zones (from 1—cool to 5—warm) based on multi‐annual mean air temperatures (1981–2010) ranging between 4.5 and 10°C. The final selection of study sites covered a spatial extent of 300 km × 300 km and 1000 m in elevation.

### 2.2 Study design and data collection

In May 2019, we established four baited pitfall traps on each of the 115 study sites. We sampled in May because the highest dung beetle diversity was expected (according to Šlachta, 2013, who sampled in April, May, June, July, and August in a similar geographic region). To attract a broad range of beetle species, we covered a trophic gradient using dung from a carnivore (Eurasian lynx, *Lynx lynx*) as the highest trophic level, an omnivore (wild boar, *Sus scrofa*) as intermediate trophic level, and two types of herbivores (red deer, *Cervus elaphus* as browser‐grazer and European bison, *Bos bonasus*, as grazer) as the lowest trophic level. We chose European bison dung because it functionally represents the current dominant domestic animal in agriculture, which is cattle. At the same time, bison is evolutionarily close to domesticated cattle. The advantage of using bison is that this species was widely distributed across Europe until the 20th century (Kuemmerle et al., 2011; Svenning, 2002), and organisms like insects could adapt to its dung. Contrary to domesticated cattle, bison prefer forests and herbaceous vegetation (Kuemmerle et al., 2011), which makes them a suitable study organism for land‐use studies like this.

The dung for the experiment was collected in March 2019 from animal enclosures; none of the defecating animals was treated with antibiotics or anthelmintics. Each dung type was thoroughly mixed to ensure uniform constituency and texture before weighing. Due to different natural appearances of dung types, dung was weighed as follows: 35 g (Eurasian lynx), 90 g (wild boar), 25 g (red deer), and 450 g (European bison).

Dung of red deer, wild boar, and lynx was put in elastic sausage nets (mesh size *c*. 1.5 cm) to avoid unintended dung dispersal on the study sites (bison heaps were heavy enough not to be removed by animals that passed through the study sites). All dung pats were stored frozen and only thawed one day before the beginning of the experiment. On the study sites, baited pitfall traps were established 5 m apart from each other. Pitfall traps (400 ml plastic cups) were filled with 200 ml liquid (70 ml propylene glycol and 130 ml water) and emptied after 14 days. Small holes beneath the rim of the cup prevented overspill in case of rain. We placed the bait in the center of a coarse mesh wire (mesh size 2 cm × 2 cm) that was placed half on the pitfall trap and half on the ground. The mesh wire and dung nets were then fixated to the ground with tent pegs. To empty the traps in the field, the coarse mesh wire with the dung was carefully removed from the pitfall traps, and the content of the pitfall traps was sifted through a tea bag paper. The tea bag containing any specimens was then put in a sampling container with 70% Ethanol. Beetles were then identified to species level by the experts and co‐authors TL, J‐AS, and DS.

The aim of this experiment was to sample all beetles that are attracted by dung, which comprises coprophagous, coprophilous, necrophilous, and copronecrophilous species (hereafter collectively referred to as dung‐visiting beetles). Necrophilous beetles were included since dung and carrion emit similar volatile organic compounds (Sladecek et al., 2021; von Hoermann et al., 2016; Weithmann et al., 2020) and attract necro‐ as well as coprophilous beetles. Hence, all species associated with this lifestyle (according to Assing & Schülke, 2012; Böhme & Lucht, 2005) were incorporated in this study (Table S1).

### 2.3 Climate variables

As climate variables, we used long‐term averages (1991–2020) of air temperature and precipitation amounts. The data for individual study plots were derived from monthly gridded observational datasets with a horizontal resolution of 1 km, from which 30‐year averages were subsequently calculated. Temperature and precipitation were only moderately correlated (Spearman's rho = −0.54, *p* < .05). The raw input datasets were provided by the German Meteorological Service (Deutscher Wetterdienst, DWD) and are described in Kaspar et al. (2013). Additionally, the local temperature was measured by dataloggers on each study site to account for small‐scale variations. However, since local temperature and multi‐annual mean temperature were highly correlated (Spearman's rho = 0.71, *p* < .05), we only included long‐term temperature data in our analysis.

### 2.4 Statistical analysis

We tested the effects of land use and climate on dung‐visiting beetle α‐diversity and local community specialization at study‐site level, and γ‐diversity among habitat and landscape types and climate zones using the software R, version 4.0.5 (R Core Team, 2021).

Alpha‐diversity on study sites was described using three metrics (data of individual traps per study site were pooled): abundance (number of individuals), species density (number of species, sensu Gotelli & Colwell, 2001), and species richness (number of species, accounting for abundance, sensu Gotelli & Colwell, 2001) (package “vegan” by Oksanen et al., 2020). We fitted a negative‐binomial generalized linear model (glm.nb) using the package “MASS” (Venables & Ripley, 2007) to provide estimates of the effects of habitat and landscape types, and temperature and precipitation data on the response variables “abundance,” “species density,” and “species richness.” Since the number of species and individuals in some samples was low, we decided against a resampling approach, such as chao1 or ACE, to calculate species richness. Instead, we accounted for abundance by including log_e_ (abundance) as a predictor in the species richness model.

Additionally, a Tukey HSD post hoc test was conducted to explore differences in abundance, species density, and species richness among habitat and landscape types (package “multcomp” by Hothorn et al., 2008). To check for potential spatial autocorrelation of the model residuals, we used cross‐correlograms (package “ncf” by Bjornstad & Falck, 2001) based on Moran's I and found no spatial autocorrelation among study sites (Figure S3).

As a measure of community specialization on dung resources at the study‐site level, the standardized two‐dimensional Shannon entropy (*H*_2_′)—ranging between 0 (no resource preference) and 1 (total specialization) (Blüthgen et al., 2006)—was calculated based on the abundance of beetle species per dung type (package “bipartite” by Dormann et al., 2009). In this framework, higher specialization translates into more exclusive use of interaction partners by the existing species, i.e., higher niche differentiation (Blüthgen, 2010). Total specialization would thus imply that each species uses only one resource. Further, *H*_2_′ calculates the interaction frequencies of two groups of different trophic levels in relation to all possible interactions, hence being network size‐independent. This makes comparisons across networks along ecological gradients possible, e.g., if species shift to a more specialized or generalized resource use with a temperature shift. In addition to *H*_2_′, the Kulback–Leibler distance *d*′ is used as an index for specialization on the species level (Blüthgen et al., 2006), which allows to identify specialization of specific dung types (lynx, boar, deer, or bison). By analogy to *H*_2_′, *d*′ ranges between 0 (no specialization) and 1 (high specialization).

After calculating *H*_2_′ (we only included study sites where at least three samples revealed dung‐visiting beetles, *n* = 94 study sites), we compared the observed *H*_2_′ values with a null model with full randomization that kept species frequencies and species richness constant (“r2dtable,” 1000 simulations). A linear model was then fitted to calculate the effects of habitat and landscape type, temperature, and precipitation on *H*_2_′ of dung‐visiting beetle communities.

In cases where one of the predictors led to a significant change in resource specialization of the dung‐visiting beetle community (*H*_2_′), we calculated the degree of specialization on individual dung types *d*′ (package “bipartite,” Dormann et al., 2009) to determine whether the community specialization *H*_2_′ resulted from specialization on a specific dung type (*d*′). Then, we fitted a linear mixed effect model to test for correlations between *d*′ and the predictor variables “dung type” and “temperature,” including “study site” as a random factor, followed by a pairwise comparison (Tukey HSD) of the specialization *d*′ between individual dung types. Consequently, we fitted a linear model including *d*′ for each dung type as response variable and “temperature” as predictor variable and plotted the results in a linear regression curve.

To test for differences in the total γ‐diversity among habitats, landscapes, and climate zones, we performed separated sample‐based rarefaction‐extrapolations (package “iNEXT,” Hsieh et al., 2020) along the Hill numbers (*q* = 0, 1, and 2) (Hill, 1973). Because Hill numbers imply mathematical properties that allow drawing conclusions about diversity across different diversity indices (Chao, Chiu, et al., 2014; Jost, 2006), there seems to be broad agreement on the use of Hill numbers to quantify species diversity (Ellison, 2010). In this approach, *q* determines the measures' sensitivity to species relative abundance, with *q* = 0 focusing on rare species (species richness), *q* = 1 focusing on common species (Shannon diversity), and the order *q* = 2 focusing on dominant species (Simpson diversity) (Chao, Gotelli, et al., 2014). Having multiple assemblages, this framework can be used to partition the Hill numbers of a pooled assemblage (γ‐diversity) into its within‐assemblage component (α‐diversity) and between‐assemblage component (β‐diversity) (Chiu et al., 2014). Allowing to weigh from rare to dominant species, this methodology seems particularly relevant in functional ecosystem engineer groups such as dung beetles, where dominant species are often the major actors in the removal process (Frank, Hülsmann, et al., 2017).

This approach is based on predictor categories, which works for our habitat and landscape types. To include climate in the Hill analysis, we used the five climate zones (1—cool, 5—warm) as described in the Section 2.1 and more detailed in Redlich et al. (2022). For each *q* and predictor variable (habitat, landscape, climate), we plotted species diversity against the number of sampling units, nonoverlapping confidence intervals indicating significant differences in γ‐diversity.

## 3 RESULTS

**FIGURE 1:**
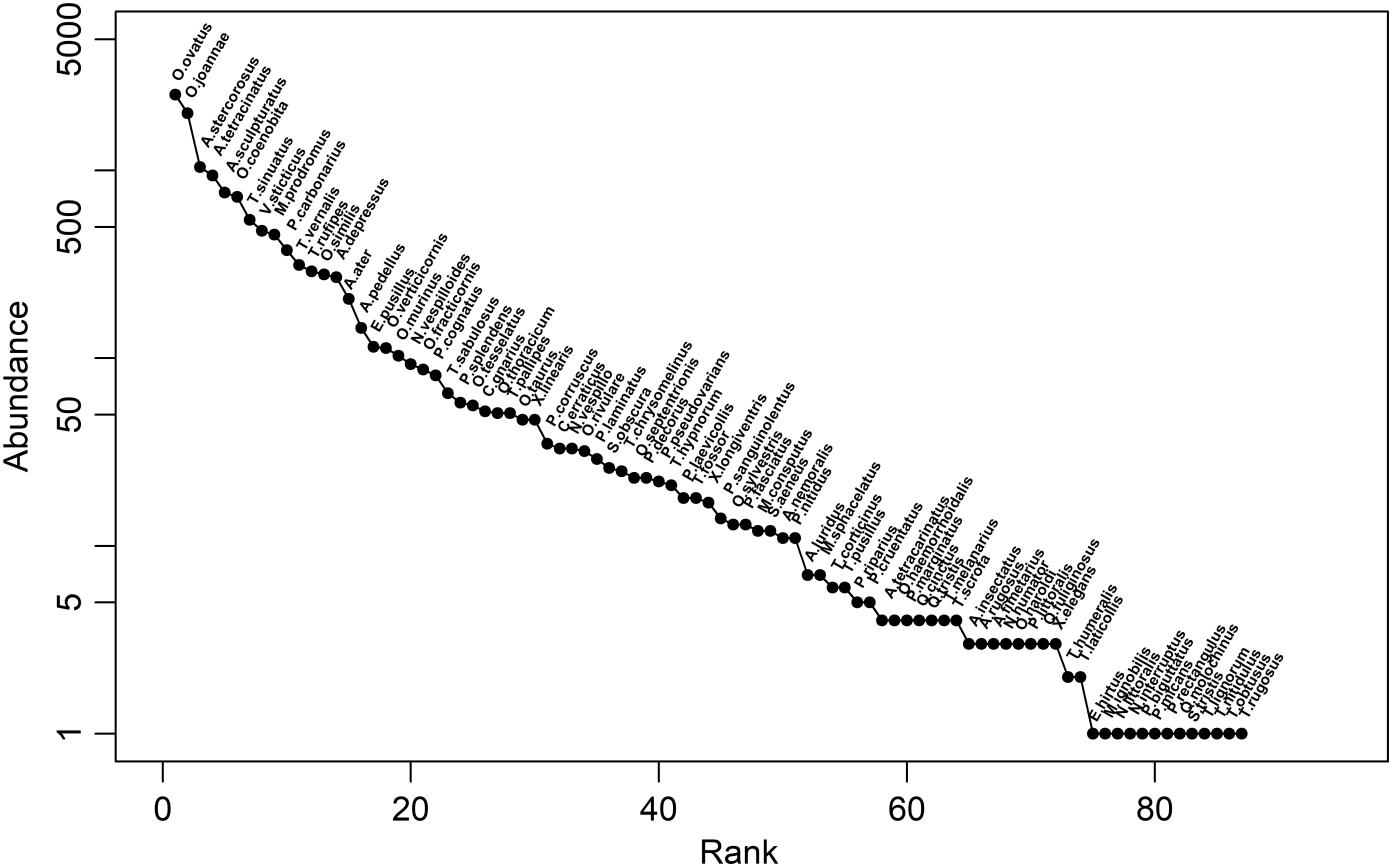
Rank abundance curve depicting the number of individuals of all recorded beetle species on a logarithmic scale

In total, 12,948 dung‐visiting beetles from 37 genera and 87 species were collected in our 385 traps. The species *Onthophagus ovatus* (Linnaeus, 1767) (Scarabaeidae) was most abundant and recorded in 62 study sites (151 traps), followed by *Onthophagus joannae* (Goljan, 1953) (Scarabaeidae) in 65 study sites (156 traps) and *Anoplotrupes stercorosus* (Hartmann in L. G. in Scriba, 1791) (Geotrupidae) in 47 study sites (113 traps) (Figure 1).

### 3.1 Alpha‐diversity

Land‐use intensity affected the abundance and species density of dung‐visiting beetles at both habitat and landscape scales (Table 1, all comparisons from post hoc test in Table S2). On the habitat scale, dung‐visiting beetle abundance was lower in arable fields compared with forest habitats (Table 1; Table S2). Species density and richness, however, were rather robust to local land use (Table S2).

**TABLE 1:** Results of the negative‐binomial generalized linear model including abundance, species density, and species richness as responses to habitat type, landscape type, and temperature and precipitation.

Predictors	Abundance			Species density			Species richness		
	Estimate	Std. error	*p*	Estimate	Std. error	*p*	Estimate	Std. error	*p*
(Intercept)	6.844	1.520	**<.001**	3.121	0.660	**<.001**	1.184	0.498	**.017**
Habitat grassland vs. forest	−0.046	0.247	.851	0.042	0.107	.693	0.057	0.070	.416
Habitat arable vs. forest	−0.651	0.254	**.010**	−0.070	0.111	.528	0.099	0.077	.199
Habitat settlement vs. forest	0.037	0.268	.891	−0.186	0.120	.122	−0.113	0.083	.176
Landscape agriculture vs. near‐natural	−0.429	0.241	.075	−0.223	0.106	**.035**	−0.093	0.073	.206
Landscape urban vs. near‐natural	0.013	0.239	.957	−0.114	0.104	.275	−0.102	0.070	.143
Temperature in °C	−0.073	0.128	0.571	−0.051	0.056	.359	−0.022	0.039	.567
Precipitation in mm	−0.002	0.001	**.013**	−0.000	0.000	.929	0.000	0.000	.107
Log(abundance)							0.305	0.026	**<.001**
Observations	115			115			115		
*R* ^2^ Nagelkerke	.157			.152			.844		

*Note*: Significant *p‐*values in bold. For complete pairwise level comparison within the categorical predictors, *p*‐values were adjusted in Table S2.

On the landscape scale, species density in agricultural landscapes was significantly lower than in near‐natural landscapes (Table 1). As expected, extending the linear analysis by pairwise tests including *p*‐value adjustment yielded a less distinctive pattern (Table S2).

Alpha‐diversity in terms of abundance, species density, and species richness was robust to temperature. Dung‐visiting beetle abundance, though, decreased with increasing precipitation (Table 1). Species richness strongly increased with increasing beetle abundance (Table 1).

### 3.2 Local community specialization on dung resources

**FIGURE 2:**
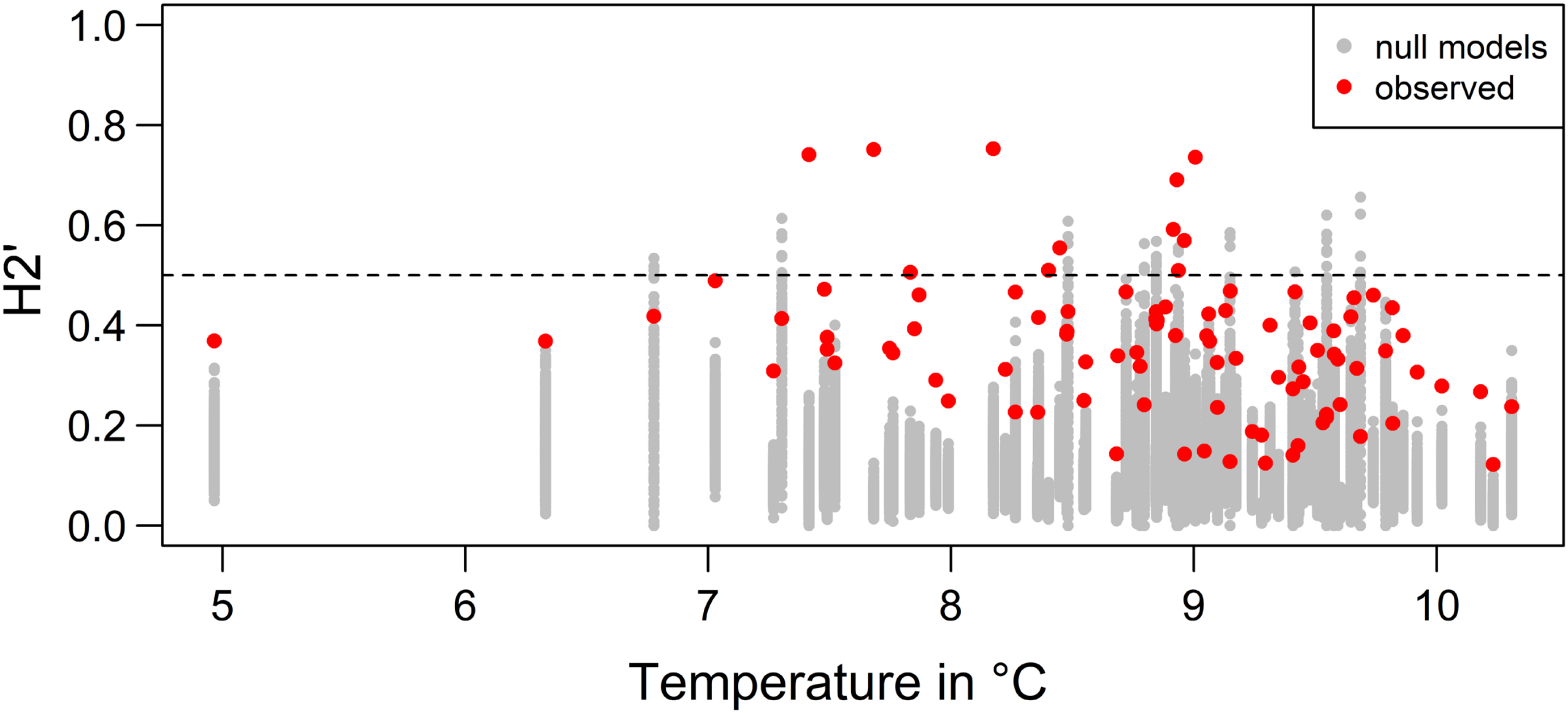
Scatterplot of observed *H*_2_′ (red dots) and randomized *H*_2_′ values (gray dots) along a mean multi‐annual temperature gradient. Dashed horizontal line indicates *H*_2_′ = 0.5. A null model calculated randomized *H*_2_′ values with 1000 simulations (in 87% of the networks, the observed *H*_2_′ was significantly higher than in random assemblages).

Excluding study sites where dung‐visiting beetles were found in less than three pitfall traps, 94 study sites (networks) were included in the analysis. The *H*_2_′ value as an index for the specialization of dung‐visiting beetle communities on dung resources did not significantly change among local habitat or regional landscape types (Table 2), and beetle assemblages in different habitats or landscapes were neither generalistic nor specialized (Figure S4). Dung‐visiting beetle assemblages did not respond to changes in precipitation but were less specialized in warmer than in cooler regions (Table 2, Figure 2).

**TABLE 2:** Results of the linear model showing the effects of habitat, landscape, temperature, and precipitation on the degree of specialization (*H*_2_′) of coprophilic beetle assemblages.

Predictors	*H*_2__obs		
	Estimates	Std. error	*p*
(Intercept)	0.765	0.237	**.002**
Habitat grassland vs. forest	0.020	0.037	.598
Habitat arable vs. forest	−0.039	0.041	.340
Habitat settlement vs. forest	0.012	0.040	.776
Landscape agriculture vs. near‐natural	0.009	0.039	.821
Landscape urban vs. near‐natural	−0.002	0.036	.953
Temperature in °C	−0.046	0.020	**.023**
Precipitation in mm	−0.000	0.000	.988
Observations	94		
*R* ^2^/*R* ^2^ adjusted	.118/.046		

*Note*: Significant *p‐*values in bold.

**FIGURE 3:**
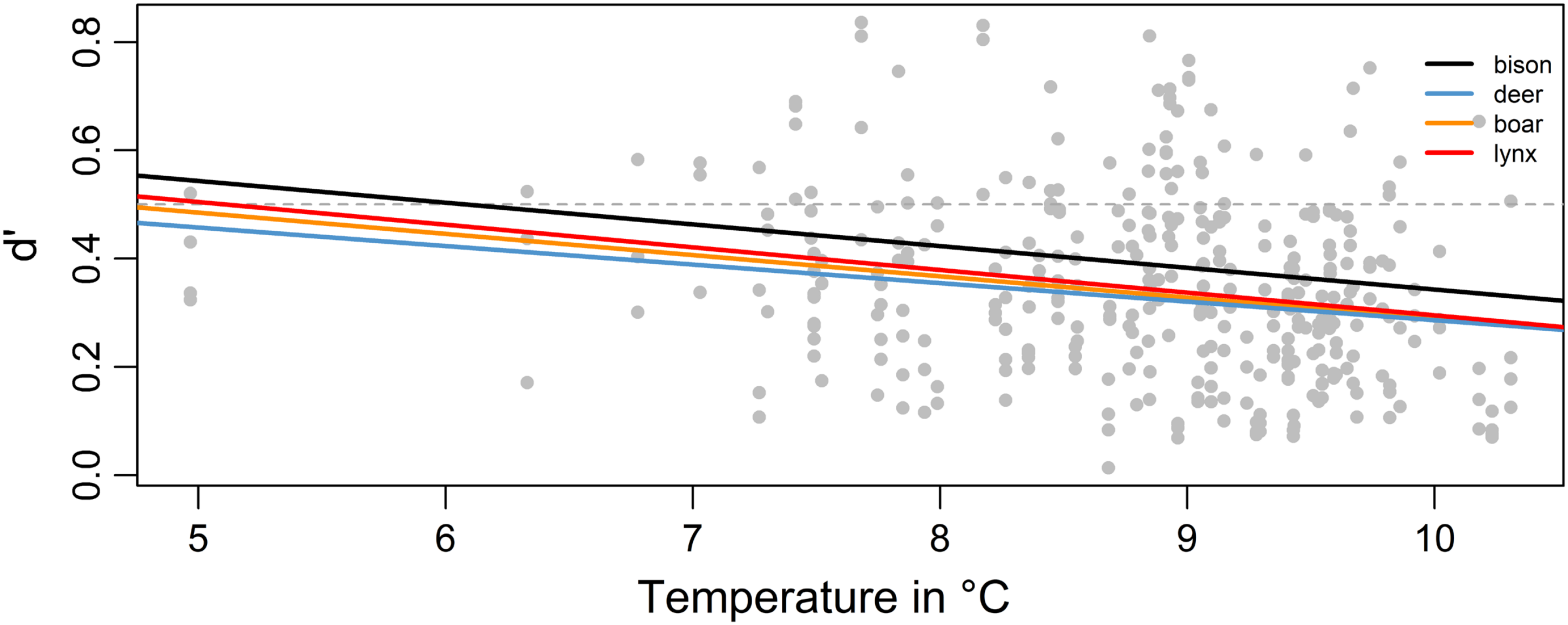
Linear regression showing the degree of specialization (*d*′) on individual dung resources along the temperature gradient. Gray dots depict individual *d*′ values; colored lines represent regression lines of each dung type. Dashed line indicates *d*′= 0.5.

Specialization (*d′*) of dung‐visiting beetles was negatively correlated with temperature and was highest for bison dung along the entire temperature gradient (Figure 3, Table 3). With cooler temperatures, specialization on bison, wild boar, and lynx dung significantly increased (Figure 3; Table S3).

**TABLE 3:** Results of the linear mixed‐effect model, testing *d*′ against temperature and dung type (study site as random effect) and Tukey HSD post hoc analysis to test for differences in specialization among dung types

Linear mixed effect model	*d*′_obs		
Predictors	Estimates	Std. error	*p*
(Intercept)	0.691	0.130	**<.001**
Deer vs. boar	0.000	0.019	1.000
Lynx vs. boar	0.012	0.018	.495
Bison vs. boar	0.057	0.018	**.002**
Multi‐annual mean temperature in °C	−0.040	0.015	**.007**
*Random effects*			
*σ* ^2^	0.01		
*τ*_00 plot_	0.01		
ICC	0.46		
*N*_plot_	94		
Observations	351		
Marginal *R* ^2^/Conditional *R* ^2^	.066/.498		

Post hoc analysis	*d*′		
	Estimate	*z*	*p*
Deer–boar	0.000	0.000	1.000
Lynx–boar	0.012	0.683	.904
Bison–boar	0.057	3.101	**.011**
Lynx–deer	0.012	0.658	.913
Bison–deer	0.060	2.987	**.015**
Bison–lynx	0.045	2.438	.070

*Note*: Significant *p‐*values in bold.

### 3.3 Gamma‐diversity

**FIGURE 4:**
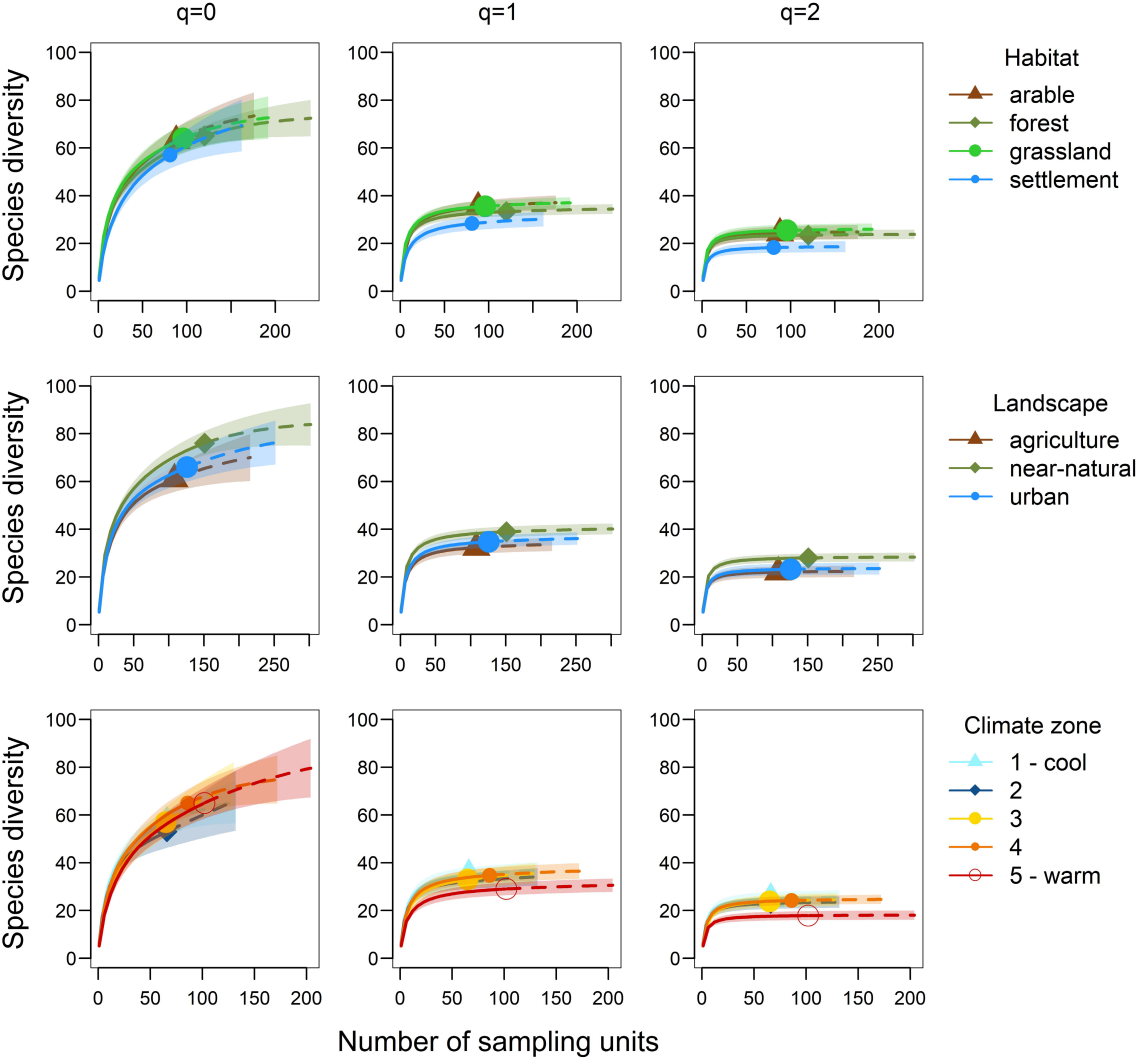
Sample size‐based rarefaction curves of rare, common, and dominant dung beetles for Hill numbers (*q* = 0, 1, and 2) across habitats, landscapes, and climate zones. Solid lines depict the interpolated number of sampling units (rarefaction), while dashed lines depict the extrapolation of sampling units. Shaded areas indicate the 95% confidence interval. Nonoverlapping confidence intervals indicate significant differences in γ‐diversity between treatments.

The rarefaction interpolation curves for *q* = 0 on the habitat scale showed no distinctive pattern for rare species diversity. At a landscape level, rare species diversity tended to be lower in agricultural and urban landscapes than in near‐natural landscapes, but this difference was not significant (Figure 4).

With increasing sensitivity to common species (*q* = 1), species diversity on a habitat scale was significantly lower in settlements than in grasslands and arable fields (Figure 4). Species diversity of common species also decreased on a landscape scale from near‐natural to urban and agricultural landscapes, with a significant difference between near‐natural and agricultural landscapes (Figure 4).

The diversity of dominant species (*q* = 2) on a habitat scale was significantly lower in settlements compared with other habitats. On a landscape scale, diversity was significantly higher in near‐natural landscapes compared with agricultural and urban landscapes (Figure 4).

The rarefaction interpolation curves showed for *q* = 0 no climate effect on rare species diversity (Figure 4). The diversity of common species (*q* = 1) in the warmest climate zone (5) was lowest with significant differences in climate zones 1 and 4 (Figure 4). Dominant species diversity (*q* = 2) was significantly lowest in the warmest climate zone compared with all other climate zones (Figure 4).

## 4 DISCUSSION

Our results provide new insights into the response of dung‐visiting beetle diversity and community specialization on dung types to land‐use intensity on local and regional scales and along a climate gradient, disentangling temperature, and precipitation. We found significant negative effects of anthropogenically transformed environments (locally and regionally) on dung‐visiting beetle abundance, species density, and γ‐diversity but not on community specialization. Climate affected dung‐visiting beetles through lower abundances associated with increasing precipitation and decreased local community specialization and γ‐diversity with higher temperatures.

### 4.1 Land‐use effects on dung beetle α‐diversity, community specialization, and γ‐diversity

In agricultural habitats and landscapes, the reduced abundance, species density, and γ‐diversity of dung‐visiting beetles might be explained by the negative effects of potentially intensified land use, including low grazing continuity, small pasture sizes, habitat fragmentation, the reduction in rangeland, and the use of pesticides that negatively impact dung beetle assemblages (Beynon et al., 2012; Buse et al., 2015; Carpaneto et al., 2007; Sánchez‐Bayo & Wyckhuys, 2019). Our findings are in line with Korasaki et al. (2013) and Carpaneto et al. (2007) and partly agree with Gebert et al. (2020), who reported land‐use effects on dung beetle abundance but not on the number of species. In our study, however, species density was significantly dependent on abundance. Hence, the reduction in species density in agricultural landscapes compared with near‐natural systems likely occurred because of lower dung‐visiting beetle abundances. This does not imply that all agricultural land use is detrimental to dung‐visiting beetles. In this case, management intensity (e.g., grazing continuity) and pasture area should be considered as important factors for the conservation of dung‐visiting beetles (Buse et al., 2015).

In settlements and to a lesser extent in urban landscapes, γ‐diversity was drastically reduced. In settlements and cities, dog dung is often the only resource for dung beetles (Carpaneto et al., 2005) due to a lack of cattle or larger wild ungulates and carnivores. The reduced variety and amount of mammalian dung can directly affect dung beetles (Errouissi et al., 2004; Iida et al., 2016; Korasaki et al., 2013; Ramírez‐Restrepo & Halffter, 2016), which potentially makes urban areas less attractive. In addition, we suggest that the amount of sealed area in settlements and cities restricts dung burying by some dung beetle species, which may limit the amount and species richness of dung burying beetles in urban environments. Dung volatiles, moreover, which attract dung beetles and guide them toward their food source (Sladecek et al., 2021), might be masked by other odors typical for urban spaces, such as car exhausts and organic waste, and might be less detectable than dung volatiles in natural environments. There is first evidence that the dung beetle *Anoplotrupes stercorosus* responds to certain plant volatiles (alpha‐pinene and camphor) (Weithmann et al., 2020), which should be lower in urban environments. If these plant volatiles are used by *A. stercorosus* for orientation or food location, however, is yet unknown (Weithmann et al., 2020) but could explain potential habitat preferences by dung beetles.

Although dung beetle species often have different habitat preferences (open versus closed habitats) (Romero‐Alcaraz & Ávila, 2000), we found no differences in α‐ and γ‐diversity between forest and grassland habitats. Findings about habitat preferences are inconsistent, though. Damborsky et al. (2015) and Frank, Hülsmann, et al. (2017) report significantly higher dung beetle richness and biomass in forests than in grasslands, whereas Romero‐Alcaraz and Ávila (2000), for instance, summarize that dung beetles are more likely to be found in open habitats. However, our forest study sites were placed within an area of tree clearing, which might be as attractive for beetles that prefer open habitats as for forest species. Moreover, study sites were not grazed during the sampling period. As a consequence, less dung was probably present, which decreases habitat quality for dung‐visiting beetles specialized on grasslands. Nonetheless, the preference for less polluted and disturbed habitats and landscapes might explain the reduced dung‐visiting beetle α‐ and γ‐diversity in agricultural and urban environments.

Dung beetle biomass (Frank, Hülsmann, et al., 2017) and multi‐species communities significantly enhance dung decomposition, even in disturbed systems (Ambrožová et al., 2021; Beynon et al., 2012; Milotić et al., 2019). Consequently, the observed lower beetle abundance, density, and γ‐diversity in urban and agricultural environments could reduce dung removal rates and disturb the balance of those ecosystems. This, in turn, might cause both ecological and economic damage (Beynon et al., 2012; Losey & Vaughan, 2006).

### 4.2 Climatic effects on α‐diversity, community specialization, and γ‐diversity

The limited occurrence of dung‐visiting beetles in regions with high precipitation might be explained by restricted flight activities (Juillet, 1964) and increased soil moisture that can be detrimental to the dung beetle larval development (Sowig, 1995; von Hoermann et al., 2020).

Unlike previous large‐scale studies by Frank et al. (2018) and Milotić et al. (2019), who report no latitudinal effect and a precipitation effect on resource specialization, respectively, we observed community specialization on dung resources with decreasing temperatures, although we only considered a temperature gradient from 5–10°C (mean annual temperature). It should be noted, though, that the networks in general were not highly specialized. The maximum *H*_2_′ value was 0.75 and only 10 out of 94 networks had *H*_2_′ bigger than 0.5, which should be considered when interpreting the results. In the large‐scale studies mentioned above, variance in community specialization was rather high, and effects might be masked by other environmental parameters, such as land use. We can confirm the assumption by Milotić et al. (2019), though, that resource specialization is linked to spatial characteristics (in temperate zones). There may be two explanations for the increased degree of specialization in cooler climates of the studied gradient: One is that the resource specialization of dung beetles is expected to increase with an increasing variety of dung resources (Frank et al., 2018). Study sites in the coolest climate zones were mainly located in, or close to, Nature and National Parks, where the density and functional diversity of larger mammals (lynx, wolf, red deer, chamois, capricorn) are higher than in other study regions, and specialization of dung‐visiting beetles is more likely. Second, dung is a nutrient‐rich but ephemeral resource that many organisms compete for. Competition is a premise for niche differentiation and specialization, both influencing community network structures and robustness (Frank et al., 2018; Frank, Brückner, et al., 2017). Dung in cold and moist regions is less prone to desiccation and persists longer than in warmer climates (Milotić et al., 2019). Hence, dung beetle species in cold climates could co‐exist by resource partitioning (McKane et al., 2002), allowing for high levels of species diversity and specialization. Since specialized communities are less robust and more prone to environmental changes and extinction (Davies et al., 2004; Neff et al., 2021), we should ensure that species in these areas will experience special consideration in conservation strategies.

We also evaluated the trophic specialization of dung‐visiting beetles among dung types. Beetles were most specialized on bison dung along the temperature gradient, although bison, as grazer, was at the bottom of our trophic gradient. Herbivorous dung (sheep dung), however, was already shown to be more attractive for dung beetles than dog dung (Carpaneto et al., 2005). Since in temperate regions more dung beetles are attracted by bigger dung heaps rather than smaller dung heaps (Errouissi et al., 2004) and dung that is available for a longer period (Buse et al., 2021), it is very likely that the high specialization on bison dung was due to its high weight, compared with the other dung types. In a global meta‐analysis, considering 45 case studies, Frank et al. (2018) found low specialization on bison dung; yet their results might be limited by the number of studies including bison dung as a research subject (*n* = 3). Since nutrient content and composition are reported to be not relevant for the long‐distance chemical attraction of dung beetles (Frank, Brückner, et al., 2017), we support the interpretation by Frank, Brückner, et al. (2017) that volatiles emitted by dung and not nutrient content might be primary determinants in dung beetles' attraction (see e.g., Weithmann et al., 2020) for what the large bison dung heaps, emitting more volatiles due to its size, were potentially more preferred despite lower nutrient content.

We show that γ‐diversity for common and dominant species was lowest in the warmest climate zone, which was to a great extent represented in NW Bavaria. This is due to Bavaria's topography with an increase in elevation from west to east and north to south, which results in a temperature gradient from warm (NW Bavaria) to cold (SE Bavaria). Since the NW corner of Bavaria is not as densely populated as other parts of our study area, we believe that the low diversity in this particular climate zone was not caused by urbanization effects. Instead, we assume that in this area there are less favorable climatic conditions for dung decomposition (Milotić et al., 2019) due to a higher risk of dung desiccation at higher temperatures. When dung desiccates and microbial activities, which are key for the emission of volatile organic compounds (Le et al., 2005), are slowed down (Anderson & Coe, 1974), consequently, insects' attraction is reduced (Davis et al., 2013). Moreover, in Harris et al. (2019) and Williams et al. (2014), increasing temperatures and low precipitation, respectively, were found to potentially decrease the abundance and diversity of some ground‐dwelling beetles. In a previous study, Englmeier et al. (2022) found a hump‐shaped pattern of dung removal rates along an elevational gradient, indicating that environmental conditions at intermediate altitudes are more beneficial for dung beetles than in lowlands. Therefore, we assume that climate warming might exacerbate the access to and decomposition of dung by insects, which could explain the potential future emigration of coprophilic beetles to cooler regions as observed by Menéndez et al. (2014).

A limiting factor in the interpretation of our results is that our sampling probably does not mirror the full dung‐visiting beetle diversity across the year, although Šlachta (2013) found the highest diversity in May.

## 5 CONCLUSION

Our study of land‐use and climate effects on diversity and community specialization of dung‐visiting beetles has shown that intensive land use (agriculture, urban areas) and climate affect dung‐visiting beetle assemblages. Diversity decreased from near‐natural to intensive land use, on a local and regional scale. Dung‐visiting beetle assemblages were more specialized in cooler climates, and hence, are likely more vulnerable to environmental changes. Our approach of a simultaneous study of climate and land use shows that both parameters affect different aspects of dung beetle communities. Urbanization and agriculture threaten diversity, while climate influences the dung specialization of communities, which might affect dung decomposition processes.

## AUTHOR CONTRIBUTIONS

**Christian von Hoermann:** Conceptualization (equal); supervision (equal); writing – original draft (equal); writing – review and editing (equal). **Daniel Rieker:** Formal analysis (equal); writing – review and editing (equal). **Marc Eric Benbow:** Conceptualization (equal); supervision (equal); writing – original draft (equal); writing – review and editing (equal). **Caryl Benjamin:** Investigation (equal); writing – review and editing (equal). **Ute Fricke:** Investigation (equal); writing – review and editing (equal). **Cristina Ganuza:** Investigation (equal); writing – review and editing (equal). **Maria Haensel:** Investigation (equal); writing – review and editing (equal). **Tomáš Lackner:** Resources (equal); writing – review and editing (equal). **Oliver Mitesser:** Formal analysis (equal); writing – review and editing (equal). **Sarah Redlich:** Conceptualization (equal); investigation (equal); project administration (equal); writing – review and editing (equal). **Rebekka Riebl:** Investigation (equal); writing – review and editing (equal). **Sandra Rojas‐Botero:** Investigation (equal); writing – review and editing (equal). **Thomas Rummler:** Resources (equal); writing – review and editing (equal). **Jörg‐Alfred Salamon:** Resources (equal); writing – review and editing (equal). **David Sommer:** Resources (equal); writing – review and editing (equal). **Ingolf Steffan‐Dewenter:** Project administration (equal); writing – review and editing (equal). **Cynthia Tobisch:** Investigation (equal); writing – review and editing (equal). **Johannes Uhler:** Investigation (equal); writing – review and editing (equal). **Lars Uphus:** Investigation (equal); writing – review and editing (equal). **Jie Zhang:** Conceptualization (equal). **Jörg Müller:** Conceptualization (equal); formal analysis (equal); methodology (equal); project administration (equal); supervision (equal); writing – original draft (equal); writing – review and editing (equal). **Jana Englmeier:** Conceptualization (lead); formal analysis (equal); methodology (lead); writing – original draft (lead); writing – review and editing (lead).

## CONFLICT OF INTEREST

The authors declare that they have no conflicts of interest.

## Supporting information


Figure S1

Figure S2

Figure S3

Figure S4

Table S1

Table S2

Table S3

Appendix S1

## Data Availability

The data that support the findings of this study are openly available in datadryad at https://doi.org/10.5061/dryad.6hdr7sr2c.
