# Supplementary figures and images for: Dung‐visiting beetle diversity is mainly affected by land use, while community specialization is driven by climate
Source: Ecol Evol. 2022 Oct 8;12(10):e9386. doi: 10.1002/ece3.9386 (PMC9547384; doi:10.1002/ece3.9386)

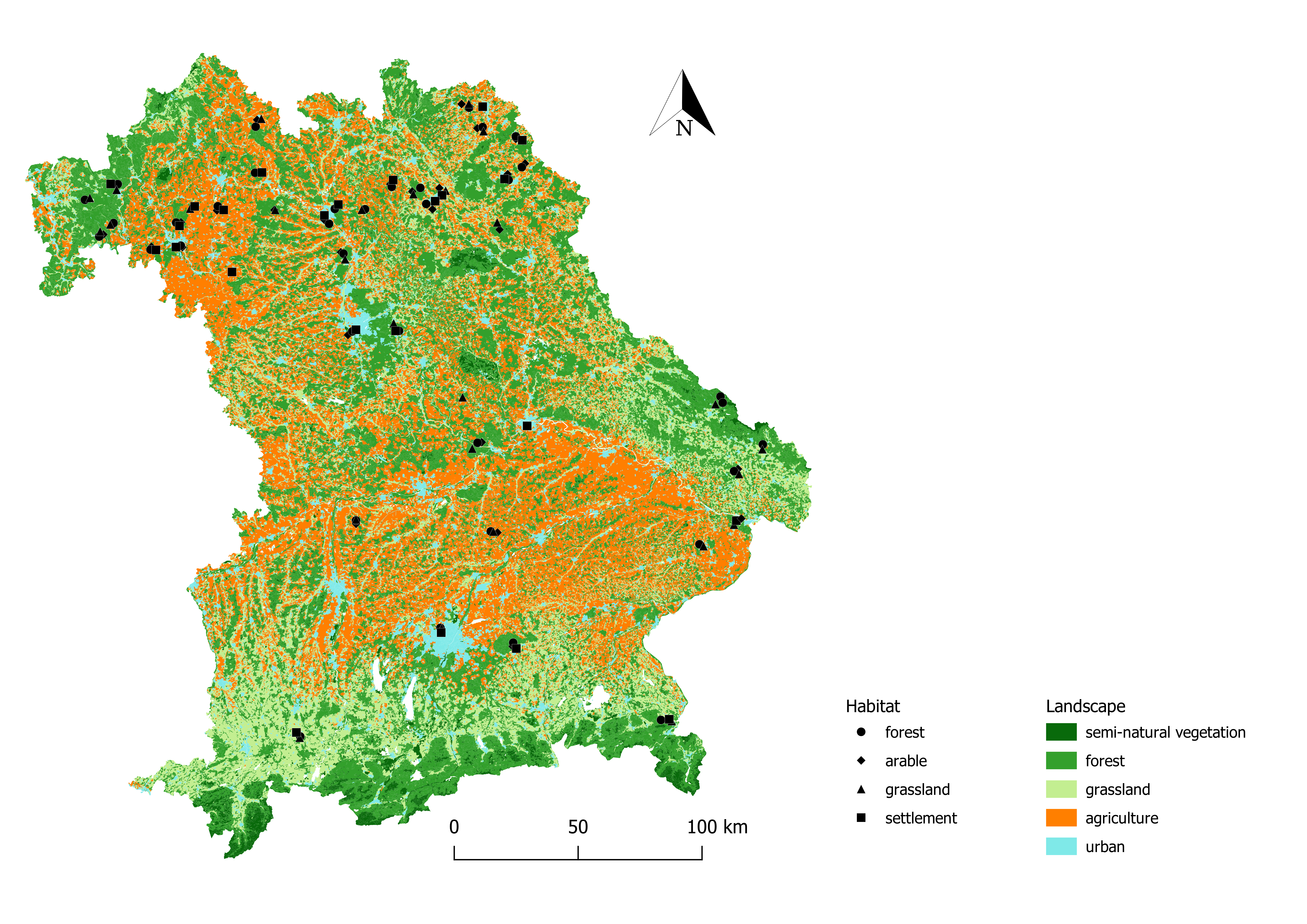

Supplement: Supplementary file 1 — Figure S1 [file ECE3-12-e9386-s006.png]

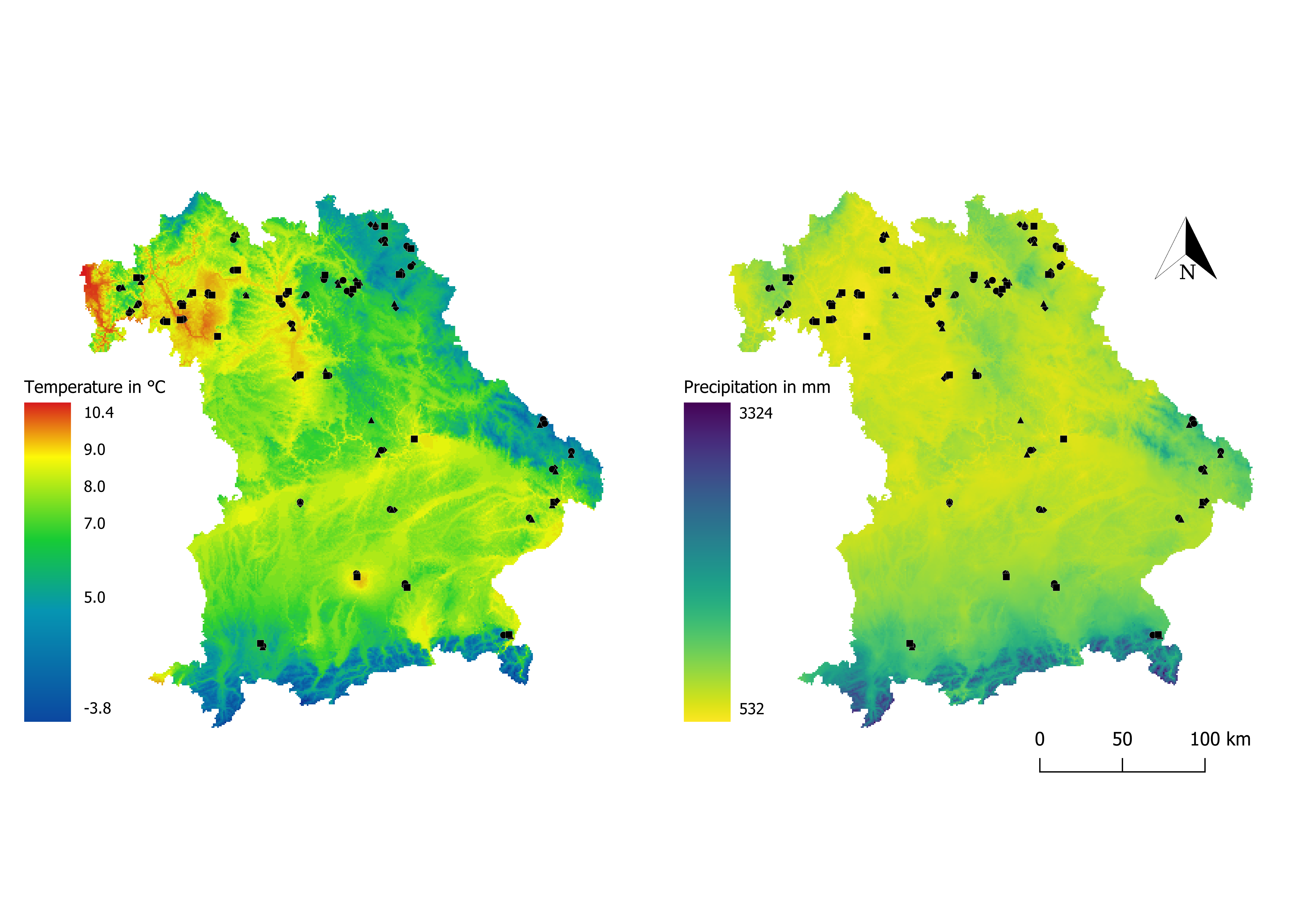

Supplement: Supplementary file 2 — Figure S2 [file ECE3-12-e9386-s007.png]

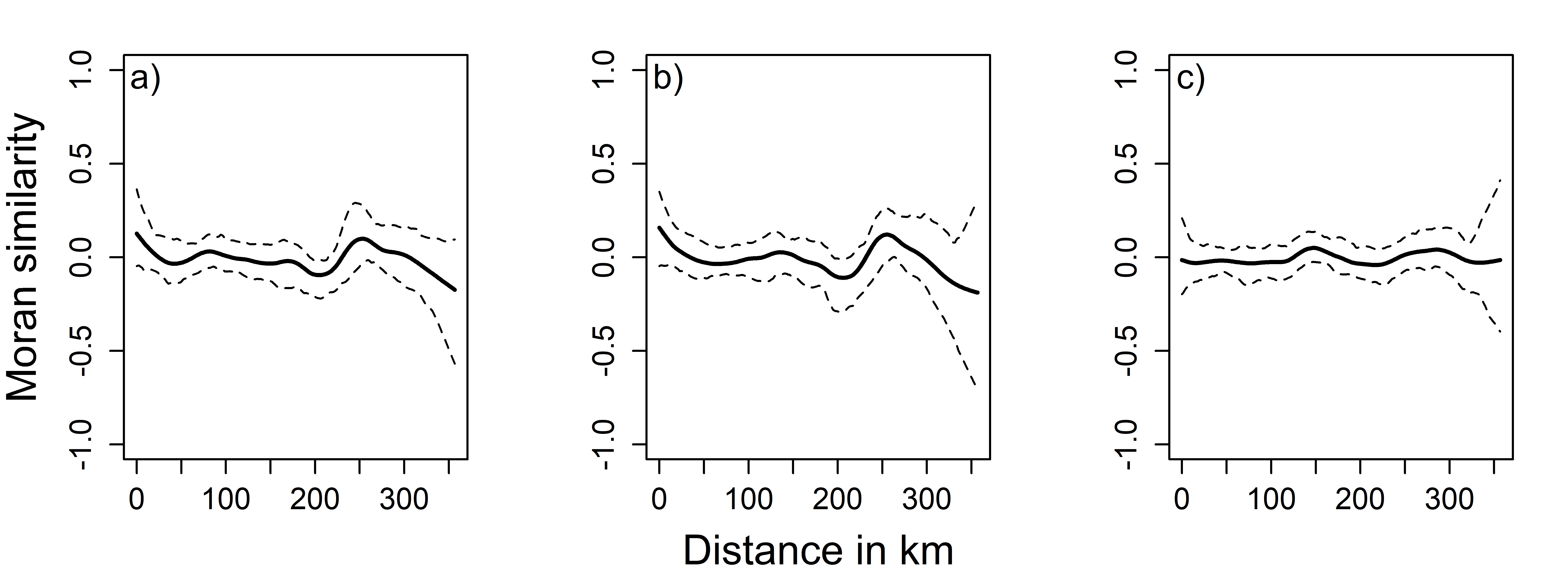

Supplement: Supplementary file 3 — Figure S3 [file ECE3-12-e9386-s005.tiff]

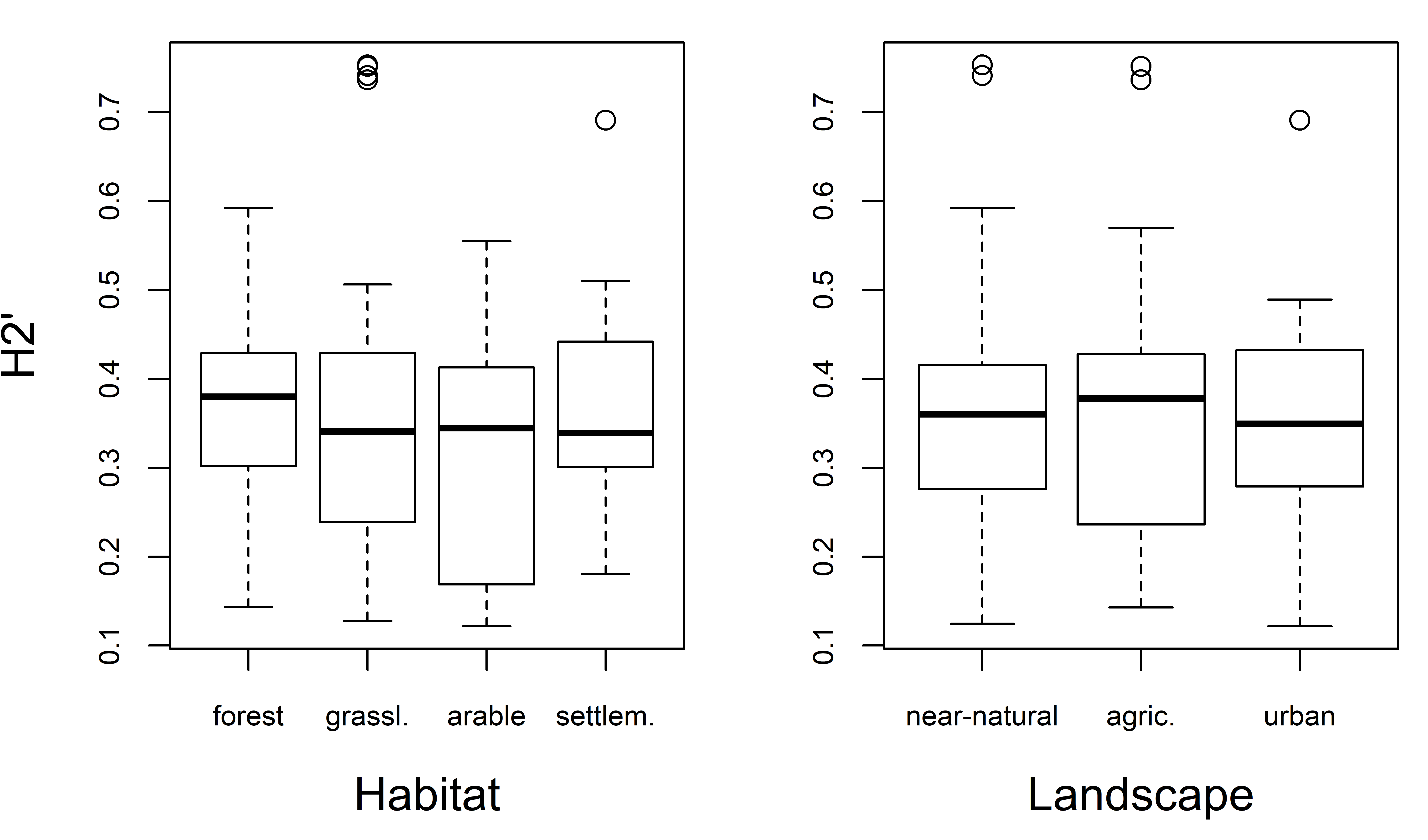

Supplement: Supplementary file 4 — Figure S4 [file ECE3-12-e9386-s001.tiff]
