# Supplementary material for: Dung‐visiting beetle diversity is mainly affected by land use, while community specialization is driven by climate
Source: Ecol Evol. 2022 Oct 8;12(10):e9386. doi: 10.1002/ece3.9386 (PMC9547384; doi:10.1002/ece3.9386)
Supplement: Supplementary file 5 — Table S1 [file ECE3-12-e9386-s003.docx]

| **Species** | **Family** | **No.** | **Diet** |
| --- | --- | --- | --- |
| *Acrossus depressus* (Kugel., 1792) | Scarabaeidae | 270 | c |
| *Acrossus luridus* (Fabricius, 1775) | Scarabaeidae | 7 | c |
| *Agoliinus nemoralis* (Erichson, 1848) | Scarabaeidae | 11 | c |
| *Agrilinus ater* (DeGeer, 1774) | Scarabaeidae | 207 | c |
| *Anoplotrupes stercorosus* (Hartmann in L. G. in Scriba, 1791) | Geotrupidae | 1042 | cn |
| *Anotylus insecatus* (Gravenhorst, 1806) | Staphylinidae | 3 | c/ phy |
| *Anotylus rugosus* (Fabricius, 1775) | Staphylinidae | 3 | c/ phy |
| *Anotylus sculpturatus* (Gravenhorst, 1806) | Staphylinidae | 763 | c/ phy |
| *Anotylus tetracarinatus* (Block, 1799) | Staphylinidae | 944 | c/ phy |
| *Aphodius fimetarius* (L., 1758) | Scarabaeidae | 3 | c |
| *Aphodius pedellus* (DeGeer, 1774) | Scarabaeidae | 145 | c |
| *Calamosternus granarius* (L., 1767) | Scarabaeidae | 52 | c |
| *Colobopterus erraticus* (L., 1758) | Scarabaeidae | 33 | c |
| *Emus hirtus* (L., 1758) | Staphylinidae | 1 | c |
| *Esymus pusillus* (Herbst, 1789) | Scarabaeidae | 115 | c |
| *Margarinotus ignobilis* (Marseul, 1854) | Histeridae | 1 | n/ ca |
| *Melinopterus consputus* (Creutzer, 1799) | Scarabaeidae | 12 | c |
| *Melinopterus prodromus* (Brahm, 1790) | Scarabaeidae | 455 | c/s |
| *Melinopterus sphacelatus* (Panzer, 1798) | Scarabaeidae | 7 | c/s |
| *Necrodes littoralis* (L., 1758) | Silphidae | 1 | n/ ca |
| *Nicrophorus humator* (Gled., 1767) | Silphidae | 3 | n/ ca |
| *Nicrophorus interruptus* (Stephens, 1830) | Silphidae | 1 | n/ ca |
| *Nicrophorus vespillo* (L., 1758) | Silphidae | 33 | n/ ca |
| *Nicrophorus vespilloides* (Herbst, 1783) | Silphidae | 93 | n/ ca |
| *Oiceoptoma thoracicum* (L., 1758) | Silphidae | 51 | n |
| *Omalium rivulare* (Paykull, 1789) | Staphylinidae | 32 | s |
| *Omalium septentrionis* (Thomson,1857) | Staphylinidae | 23 | s |
| *Ontholestes haroldi* (Eppelsheim, 1884) | Staphylinidae | 3 | c |
| *Ontholestes murinus* (L., 1758) | Staphylinidae | 103 | n/c |
| *Ontholestes tesselatus* (Geoffr., 1785) | Staphylinidae | 56 | c/n |
| *Onthophagus coenobita* (Herbst, 1783) | Scarabaeidae | 722 | c/ne |
| *Onthophagus fracticornis* (Preyssler, 1790) | Scarabaeidae | 87 | c/ne |
| *Onthophagus joannae* (Goljan, 1953) | Scarabaeidae | 2020 | c |
| *Onthophagus ovatus* (L., 1767) | Scarabaeidae | 2537 | c/ne |
| *Onthophagus similis* (L. G. Scriba, 1790) | Scarabaeidae | 279 | c/ne |
| *Onthophagus taurus* (Schreber, 1759) | Scarabaeidae | 47 | c |
| *Onthophagus verticicornis* (Laich., 1781) | Scarabaeidae | 113 | c |
| *Otophorus haemorrhoidalis* (L., 1758) | Scarabaeidae | 4 | c |
| *Oxyomus sylvestris* (Scopoli, 1763) | Scarabaeidae | 13 | c |
| *Paederus littoralis* (Gravenhorst, 1802) | Staphylinidae | 3 | ca/ phytophagous |
| *Paederus riparius* (L., 1758) | Staphylinidae | 5 | ca/ phytophagous |
| *Phalacronothus biguttatus* (Germar, 1824) | Scarabaeidae | 1 | c |
| *Philonthus carbonarius* (Gravenhorst, 1802) | Staphylinidae | 376 | ca |
| *Philonthus cognatus* (Stephens, 1832) | Staphylinidae | 81 | ca |
| *Philonthus corruscus* (Gravenhorst, 1802) | Staphylinidae | 35 | ca |
| *Philonthus cruentatus* (Gmelin, 1790) | Staphylinidae | 5 | ca |
| *Philonthus decorus* (Gravenhorst, 1802) | Staphylinidae | 23 | ca |
| *Philonthus laevicollis* (Lacordaire, 1835) | Staphylinidae | 18 | ca |
| *Philonthus laminatus* (Creutzer, 1799) | Staphylinidae | 29 | ca |
| *Philonthus marginatus* (O. F. Müller, 1764) | Staphylinidae | 4 | c/ca |
| *Philonthus micans* (Gravenhorst, 1802) | Staphylinidae | 1 | ca |
| *Philonthus nitidus* (F., 1787) | Staphylinidae | 11 | c/ca |
| *Philonthus pseudovarians* (A. Strand, 1941) | Staphylinidae | 22 | ca |
| *Philonthus rectangulus* (Sharp, 1874) | Staphylinidae | 1 | ca |
| *Philonthus sanguinolentus* (Gravenhorst, 1802) | Staphylinidae | 14 | ca |
| *Philonthus splendens* (F., 1792) | Staphylinidae | 58 | c/ca |
| *Planolinus fasciatus* (Olivier, 1789) | Scarabaeidae | 13 | c |
| *Quedius cinctus* (Payk., 1790) | Staphylinidae | 4 | c/ca |
| *Quedius fuliginosus* (Gravenhorst, 1802) | Staphylinidae | 3 | ca |
| *Quedius molochinus* (Gravenhorst, 1806) | Staphylinidae | 1 | ca |
| *Quedius tristis* (Gravenhorst, 1802) | Staphylinidae | 4 | ca |
| *Saprinus aeneus* (F., 1775) | Histeridae | 12 | n/ca |
| *Silpha obscura* (L., 1758) | Silphidae | 26 | n/e |
| *Silpha tristis* (Illiger, 1798) | Silphidae | 1 | n/e |
| *Tachinus corticinus* (Gravenhorst, 1802) | Staphylinidae | 6 | s (on dung, fungi and carrion) |
| *Tachinus humeralis* (Gravenhorst, 1802) | Staphylinidae | 2 | s (on dung, fungi and carrion) |
| *Tachinus laticollis* (Gravenhorst, 1802) | Staphylinidae | 2 | s (on dung, fungi and carrion) |
| *Tachinus lignorum* (L., 1758) | Staphylinidae | 1 | s (on dung, fungi and carrion) |
| *Tachinus pallipes* (Gravenhorst, 1806) | Staphylinidae | 51 | s (on dung, fungi and carrion) |
| *Tachinus rufipes* (L., 1758) | Staphylinidae | 290 | s (on dung, fungi and carrion) |
| *Tachyporus chrysomelinus* (L., 1758) | Staphylinidae | 25 | ca |
| *Tachyporus hypnorum* (F., 1775) | Staphylinidae | 21 | ca |
| *Tachyporus nitidulus* (F., 1781) | Staphylinidae | 1 | ca |
| *Tachyporus obtusus* (L., 1767) | Staphylinidae | 1 | ca |
| *Tachyporus pusillus* (Gravenhorst, 1806) | Staphylinidae | 6 | ca |
| *Tasgius melanarius melanarius* (Heer, 1839) | Staphylinidae | 4 | ca |
| *Teuchestes fossor* (L., 1758) | Scarabaeidae | 18 | c |
| *Thanatophilus rugosus* (L., 1758) | Silphidae | 1 | n |
| *Thanatophilus sinuatus* (F., 1775) | Silphidae | 546 | n |
| *Trichonotulus scrofa* (F., 1787) | Scarabaeidae | 4 | c |
| *Trox sabulosus* (L., 1758) | Trogidae | 65 | n |
| *Trypocopris vernalis* (L., 1758) | Geotrupidae | 314 | c/cn |
| *Volinus sticticus* (Panzer, 1798) | Scarabaeidae | 477 | c |
| *Xantholinus elegans* (Oliver, 1795) | Staphylinidae | 3 | ca |
| *Xantholinus linearis* (Oliver, 1795) | Staphylinidae | 47 | ca |
| *Xantholinus longiventris* (Heer, 1839) | Staphylinidae | 17 | ca |
| c: coprophagous; ca: carnivorous; cn: copronecrophagous; e: entomophagous; n: necrophagous; ne: necrophilous; phy: phytosaprophagous; s: saprophagous | | | |
