# Supplementary material for: Dung‐visiting beetle diversity is mainly affected by land use, while community specialization is driven by climate
Source: Ecol Evol. 2022 Oct 8;12(10):e9386. doi: 10.1002/ece3.9386 (PMC9547384; doi:10.1002/ece3.9386)
Supplement: Supplementary file 6 — Table S2 [file ECE3-12-e9386-s002.docx]

| **Post-hoc** | **Abundance** | | | **Species density** | | | **Species richness** | | |
| --- | --- | --- | --- | --- | --- | --- | --- | --- | --- |
| *Habitat* | *Estimate* | *z* | *p* | *Estimate* | *z* | *p* | *Estimate* | *z* | *p* |
| grassland- forest | -0.046 | -0.188 | 0.998 | 0.042 | 0.394 | 0.979 | 0.057 | 0.814 | 0.847 |
| arable - forest | -0.651 | -2.565 | 0.050 | -0.070 | -0.630 | 0.922 | 0.099 | 1.284 | 0.571 |
| settlement- forest | 0.034 | 0.138 | 0.999 | -0.186 | -1.547 | 0.408 | -0.113 | -1.354 | 0.526 |
| arable - grassland | -0.604 | -2.256 | 0.108 | -0.112 | -0.961 | 0.771 | 0.042 | 0.522 | 0.953 |
| settlement- grassland | 0.083 | 0.294 | 0.991 | -0.228 | -1.811 | 0.267 | -0.170 | -1.968 | 0.198 |
| settlement- arable | 0.688 | 2.431 | 0.071 | -0.116 | -0.909 | 0.799 | -0.211 | -2.379 | 0.080 |
|  |  |  |  |  |  |  |  |  |  |
| *Landscape* |  |  |  |  |  |  |  |  |  |
| agriculture - near-natural | -0.430 | -1.780 | 0.176 | -0.223 | -2.105 | 0.089 | -0.093 | -1.264 | 0.415 |
| urban - near-natural | 0.013 | 0.054 | 0.998 | -0.114 | -1.092 | 0.519 | -0.102 | -1.464 | 0.308 |
| urban - agriculture | 0.442 | 1.858 | 0.151 | 0.109 | 1.022 | 0.563 | -0.009 | -0.126 | 0.991 |
