# Supplementary material for: Dung‐visiting beetle diversity is mainly affected by land use, while community specialization is driven by climate
Source: Ecol Evol. 2022 Oct 8;12(10):e9386. doi: 10.1002/ece3.9386 (PMC9547384; doi:10.1002/ece3.9386)
Supplement: Supplementary file 7 — Table S3 [file ECE3-12-e9386-s004.docx]

|  | **d' of bison** | | | **d' of deer** | | |
| --- | --- | --- | --- | --- | --- | --- |
| *Predictors* | *Estimates* | *std. Error* | *p* | *Estimates* | *std. Error* | *p* |
| (Intercept) | 0.804 | 0.170 | **<0.001** | 0.491 | 0.195 | **0.014** |
| Temperature in °C | -0.047 | 0.019 | **0.017** | -0.019 | 0.022 | 0.394 |
| Observations | 87 | | | 81 | | |
| R^2^ / R^2^ adjusted | 0.065 / 0.054 | | | 0.009 / -0.003 | | |
|  | **d' of boar** | | | **d’ of lynx** | | |
| *Predictors* | *Estimates* | *std. Error* | *p* | *Estimates* | *std. Error* | *p* |
| (Intercept) | 0.663 | 0.161 | **<0.001** | 0.772 | 0.167 | **<0.001** |
| Temperature in °C | -0.037 | 0.018 | **0.044** | -0.048 | 0.019 | **0.012** |
| Observations | 91 | | | 92 | | |
| R^2^ / R^2^ adjusted | 0.045 / 0.034 | | | 0.068 / 0.058 | | |
