# Supplementary material for: Dung‐visiting beetle diversity is mainly affected by land use, while community specialization is driven by climate
Source: Ecol Evol. 2022 Oct 8;12(10):e9386. doi: 10.1002/ece3.9386 (PMC9547384; doi:10.1002/ece3.9386)
Supplement: Supplementary file 8 — Appendix S1 [file ECE3-12-e9386-s008.docx]

**Figures and Tables**

Figure1: Rank abundance curve depicting the number of individuals of all recorded beetle species on a logarithmic scale.

Figure 2: Scatterplot of observed H_2_' (red dots) and randomized H_2_' values (grey dots) along a mean multi-annual temperature gradient. Dashed horizontal line indicates H_2_' = 0.5. A null-model calculated randomized H2' values with 1000 simulations (in 87 % of the networks, the observed H_2_' was significantly higher than in random assemblages).

Figure 3: Linear regression showing the degree of specialization (d') on individual dung resources along the temperature gradient. Grey dots depict individual d' values, colored lines represent regression lines of each dung type. Dashed line indicates d' = 0.5.

Figure 4: Sample-size based rarefaction curves of rare, common, and dominant dung beetles for Hill numbers (*q* = 0, 1, and 2) across habitats, landscapes, and climate zones. Solid lines depict the interpolated number of sampling units (rarefaction), while dashed lines depict the extrapolation of sampling units. Shaded areas indicate the 95% confidence interval. Non-overlapping confidence intervals indicate significant differences in γ-diversity between treatments.

Table 1: Results of the negative-binomial generalized linear model including abundance, species density, and species richness as responses to habitat type, landscape type, and temperature and precipitation. Significant *p-*values in bold. For complete pairwise level comparison within the categorical predictors *p*-values were adjusted in Table A2.

Table 2: Results of the linear model showing the effects of habitat, landscape, temperature and precipitation on the degree of specialization (H_2_') of coprophilic beetle assemblages. Significant *p-*values in bold.

Table 3: Results of the linear mixed effect model, testing d’ against temperature and dung type (study site as random effect) and TukeyHSD post-hoc analysis to test for differences in specialization among dung types. Significant *p-*values in bold.

**Appendix**

Figure A1: Location of the 115 study sites within the land-use gradient (categories according to CORINE, 2012. Habitat types are depicted as black symbols.

Figure A2: Location of the 115 study sites along the temperature (left) and precipitation (right) gradients in Bavaria, Germany. The temperature- and precipitation-gradients comprise 30-year annual means (1981-2010).

Figure A3: Cross-correlogram of the negative-binomial generalized linear model residuals for a) abundance, b) species density, and c) species richness

Figure A4: Boxplots indicating the degree of specialization (H_2_') in different habitats and landscapes

Table A1: Full species list of necro-/coprophilic beetles (according to Böhme & Lucht, 2005 Assing & Schülke, 2012) trapped in our experiment and included in the analysis

Table A2: Post-hoc test comparing abundance, species density, and species richness among habitats and landscapes.

Table A3: Linear model output testing d' against temperature for individual dung types. Significant *p-*values in bold.
